# Leptin signaling enhances cell invasion and promotes the metastasis of human pancreatic cancer via increasing MMP-13 production

**DOI:** 10.18632/oncotarget.3878

**Published:** 2015-04-19

**Authors:** Yingchao Fan, Yu Gan, Yuling Shen, Xiaojin Cai, Yanfang Song, Fangyu Zhao, Ming Yao, Jianren Gu, Hong Tu

**Affiliations:** ^1^ State Key Laboratory of Oncogenes and Related Genes, Shanghai Cancer Institute, Renji Hospital, Shanghai Jiao Tong University School of Medicine, Shanghai, China; ^2^ Department of Head and Neck Surgery, Renji Hospital, Shanghai Jiao Tong University School of Medicine, Shanghai, China

**Keywords:** leptin, metastasis, MMP-13, Ob-Rb, pancreatic cancer

## Abstract

Emerging evidence has suggested that leptin, an adipokine related to energy homeostasis, plays a role in cancer growth and metastasis. However, its impact on pancreatic cancer is rarely studied. In this study, we found that leptin's functional receptor Ob-Rb was expressed in pancreatic cancer cell lines. Treatment with leptin enhanced the migration and invasion of pancreatic cancer cells but did not affect the proliferation of human pancreatic cancer cells. Leptin up-regulated the expression of matrix metalloproteinase-13 (MMP-13) via the JAK2/STAT3 signaling pathway. The overexpression of leptin was shown to significantly promote tumor growth and lymph node metastasis in a subcutaneous model and an orthotopic model of human pancreatic cancer, respectively. Furthermore, in human pancreatic cancer tissues, the expression of Ob-Rb was positively correlated with the MMP-13 level. The increased expression of either Ob-Rb or MMP-13 was significantly associated with lymph node metastasis and tended to be associated with the TNM stage in patients with pancreatic cancer. Our findings suggest that leptin enhances the invasion of pancreatic cancer through the increase in MMP-13 production, and targeting the leptin/MMP-13 axis could be an attractive therapeutic strategy for pancreatic cancer.

## INTRODUCTION

Pancreatic cancer is one of the most malignant neoplasms among all gastrointestinal cancers, with a 5-year survival rate below 6% and a median survival period of less than 6 months [1, 2]. In the past decades, none of the novel approaches have added meaningful clinical or survival benefits in this patient population [3]. The lack of effective therapy is partly due to the aggressive tumor biology, but also reflects the current insufficient understanding of the mechanisms of pancreatic cancer growth and dissemination.

Etiological studies have provided evidence supporting that obesity and diabetes are independent risk factors for developing pancreatic cancer [4-6]. Obesity has also been associated with increased lymph node (LN) metastasis in patients with resected pancreatic cancer [7]. Moreover, both obesity and diabetes have been associated with decreased survival among pancreatic cancer patients [7, 8]. However, the mechanisms underlying their influence on pancreatic cancer development and progression remain poorly understood. Obesity-induced alterations in the adipocytokine milieu have been linked to several systemic diseases [9]. Leptin is one of the most studied adipocytokines, and its peripheral level has been demonstrated to be substantially increased in obese humans [10, 11], as well as in patients with diabetes [12]. As an important adipocyte-derived peptide hormone, leptin functions in regulating food intake and energy metabolism [13]. It is also produced by non-adipose tissues [14], and may function in the modulation other biological processes, such as angiogenesis, hematopoiesis, reproduction, and bone formation [15-17]. Importantly, emerging evidence suggests that leptin plays an important role in cancer proliferation, invasion, and metastasis [18]. Given that obesity and diabetes have been associated with the development of numerous cancers [19, 20], leptin may act as a link between metabolic disorders and cancer.

The role of leptin signaling in cancer has been explored in various studies. Leptin exerts its biological activity through its specific receptor (Ob-R), which belongs to the class I cytokine receptor family [21]. Although there are at least six receptor isoforms that have been identified, only the full long form of the leptin receptor (Ob-Rb) contains the intracellular motifs necessary for the initiation of intracellular leptin signaling [21]. Ob-Rb was detected in diverse cell lines derived from various cancers, such as liver cancer [22], ovarian cancer [23], colorectal cancer [24], and cholangiocarcinoma [25]. Additionally, treatment with leptin was shown to significantly stimulate the proliferation and/or invasion of malignant cells *in vitro* [22-26]. Moreover, the growth of colon tumors was substantially retarded in leptin-deficient (*ob*/*ob*) or leptin receptor-deficient (*db*/*db*) mice [27], suggesting an important role for leptin signaling in tumor regulation *in vivo*.

A recent study reported that the leptin receptor was detected in pancreatic cancer cells, and hypoxia inducible factor (HIF)-1α could directly regulate its expression [28]. However, the biological consequences and molecular mechanisms underlying the activation of the leptin signaling pathway in pancreatic cancer cells have not been investigated in detail. In this study, we investigated the role of leptin signaling in pancreatic cancer cells through *in vitro* and *in vivo* studies. More importantly, we assessed the relationship between Ob-Rb and the clinicopathological characteristics of pancreatic cancer.

## RESULTS

### Leptin promotes the invasion and migration, but not the proliferation, of pancreatic cancer cells *in vitro*

**Figure 1:**
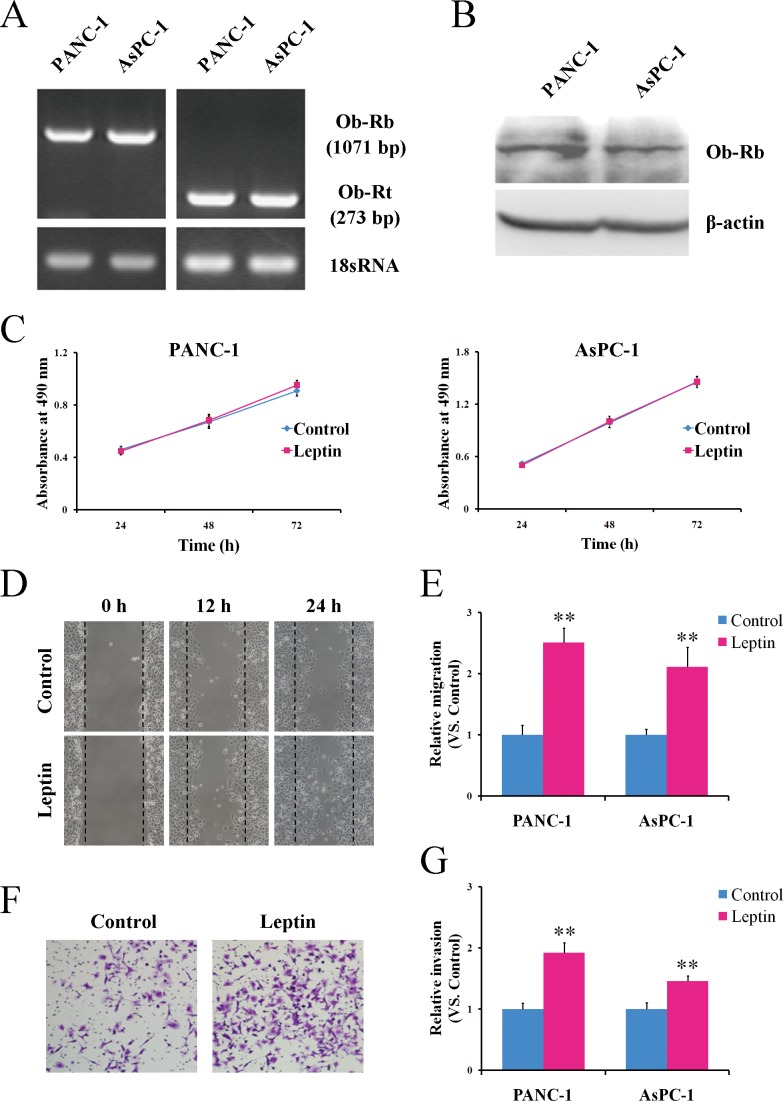
Leptin promotes the migration and invasion potential of human pancreatic cancer cells *in vitro* **A.** The total RNA was extracted from PANC-1 and AsPC-1 cells and analyzed via semiquantitative reverse transcription-PCR using specific primers for Ob-Rt and Ob-Rb. The primers for 18S RNA were used as a control. **B.** The total protein was isolated from PANC-1 and AsPC-1 and was subjected to a western blot analysis of Ob-Rb. **C.** The PANC-1 and AsPC-1 cells were left untreated or were treated with leptin for the indicated time durations, and the cell proliferation was then determined via MTS assay. The results are expressed as the absorbance at 490 nm. **D.** The PANC-1 and AsPC-1 cells were left untreated or were treated with leptin for 24 h and were subjected to a scratch assay. The cells were photographed at the indicated time points after the cell scratching. **E.** The scratch-induced cell migration was quantitated as described in the Materials and Methods. The histogram represents the quantitative analysis of the cell migration at 24 h after the cell scratching. **F.** Untreated or leptin-treated PANC-1 and AsPC-1 cells were cultured in Matrigel-coated chambers for 24 h. The cells that invaded through the Matrigel were stained using crystal violet and photographed. **G.**The cell invasion was quantitated by counting the invaded cells. Leptin promoted the migration and invasion of the pancreatic cancer cells *in vitro*. The data are presented as the means ± SEM of 4 independent experiments. ** *P* < 0.01, compared with the untreated cells.

Because leptin exerts its biological effects via binding to specific receptors [21], we first determined whether leptin receptors (Ob-Rs) existed in the human pancreatic cancer cells PANC-1 and AsPC-1. The Ob-Rs mRNA was measured via reverse transcription PCR (RT-PCR) using specific primers targeting the total isoforms (Ob-Rt) and the full long isoform (Ob-Rb) of the leptin receptor. As shown in Figure 1A, the predicted PCR products of Ob-Rt (273 bp) and Ob-Rb (1,071 bp) were observed in both the PANC-1 and AsPC-1 cells. At the protein level, Ob-Rb was detected via western blotting using the antibody that recognizes only the long form of the leptin receptor (Figure 1B). These results confirmed that Ob-Rb, a functional receptor responsible for intracellular signal transduction [21], is present in pancreatic cells.

We next investigated the influence of leptin on the proliferation of human pancreatic cancer cells. Serum-starved PANC-1 and AsPC-1 cells were treated with 100 ng/ml of recombinant human leptin. At this concentration, leptin has been reported to significantly stimulate the growth of various cancer cells [22, 23, 25, 29]. However, as shown in Figure 1C, the treatment with leptin had no influence on the proliferation of either the PANC-1 or AsPC-1 cells.

As both the migration and invasion of tumor cells contribute to the metastasis of pancreatic cancer [30], we next addressed whether leptin could influence the migration and invasion potential of human pancreatic cancer cells. Using a scratch assay, the numbers of both the PANC-1 (*P* < 0.01) and AsPC-1 cells (*P* < 0.01) that migrated to the scratched area were greater in the cells treated with leptin than in those without leptin treatment (Figure 1D). Additionally, the leptin significantly accelerated the invasion of the pancreatic cancer cells through a Matrigel-reconstituted basement membrane matrix towards the bottom chamber (Figure 1E). Crystal-violet staining of the invaded cells exhibited significant invasions of both the PANC-1 (*P* < 0.01) and the AsPC-1 cells (*P* < 0.01) in response to the leptin treatment (Figure 1F). Collectively, our data suggest that leptin can promote the migration and invasion of human pancreatic cancer cells but has no effect on cell proliferation.

### Leptin activates the JAK2/STAT3 signaling pathway in the enhancement of the migration and invasion of pancreatic cancer cells

**Figure 2:**
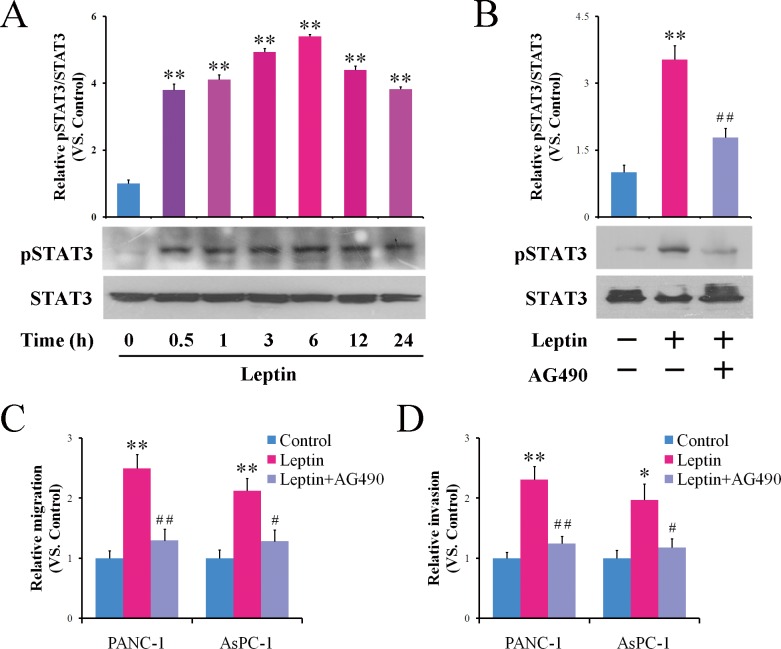
Leptin enhances the migration and invasion of pancreatic cancer cells via activating JAK2/STAT3 signaling **A.** PANC-1 cells were treated with leptin for the indicated time interval. Time 0 represents the absence of leptin or the untreated cells. The cell lysates were prepared and subjected to a western blotting analysis using specialized antibody against the total or phosphorylated forms of STAT3. The histogram shows the densitometric analysis of the bands showing a significant increase in the levels of the phosphorylated forms of STAT3 with respect to the total protein. **B**-**D.** PANC-1 or AsPC-1 cells were treated with leptin alone or in combination with the JAK2 inhibitor AG490 for 24 h. The phosphorylation of STAT3 was analyzed via western blot **B.**. The treated cells were also subjected to an *in vitro* scratch assay **C.** and a Matrigel-based invasion assay **D.**. Blocking of the JAK2/STAT3 signaling attenuated the leptin-stimulated migration and invasion of the pancreatic cancer cells. The data are presented as the means ± SEM of 3-4 independent experiments. * *P* < 0.05, ** *P* < 0.01, compared with the untreated control cells; ^#^ *P* < 0.05, ^##^ *P* < 0.01, compared with the leptin-treated cells.

The intracellular signaling of leptin is considered to be primarily transmitted through the JAK/STAT pathway [31]. Therefore, we examined whether the JAK/STAT signal pathway is also involved in leptin's action in pancreatic cancer cells. Total protein lysates of PANC-1 cells treated with leptin for various time periods were collected to detect the phosphorylation level of STAT3. As shown in Figure 2A, leptin stimulated the phosphorylation of STAT3 in a time-dependent manner. The phosphorylated STAT3 (pSTAT3) was increased significantly during the first 30 min and was maintained for at least 24 h after the treatment (Figure 2A).

We then tested the effect of the JAK2 inhibitor AG490 on the leptin-induced enhancement of the migration and invasion of the pancreatic cancer cells. The treatment of the cells with AG490 significantly decreased the intracellular level of pSTAT3 stimulated by leptin (Figure 2B). Accordingly, blocking the STAT3 phosphorylation significantly attenuated the enhancement of the migration (Figure 2C) and invasion (Figure 2D) induced by leptin in both the PANC-1 and AsPC-1 cells. These data suggest the involvement of the JAK2/STAT3 pathway in the leptin-induced enhancement of migration and invasion in pancreatic cancer cells.

### Matrix metalloproteinase 13 (MMP-13) serves as a downstream effector of the leptin -JAK2/STAT3 cascade responsible for cell invasion in pancreatic cancer cells

**Figure 3:**
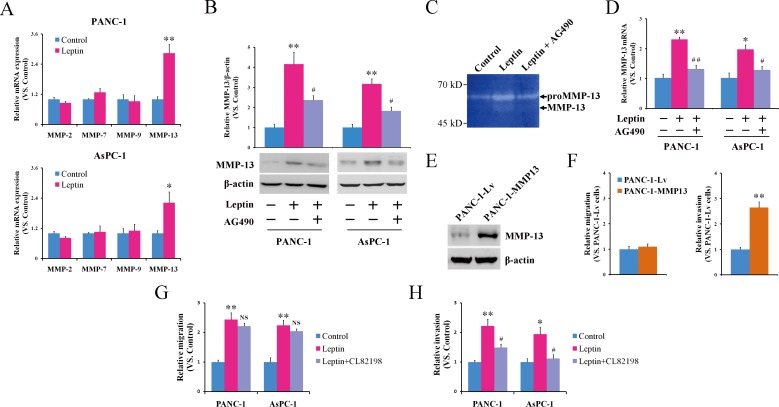
MMP-13 acts as a downstream effector of JAK2/STAT3 activation and cell invasion stimulated by leptin **A.** PANC-1 and AsPC-1 cells were left untreated or were treated with leptin for 24 h, followed by a quantitative reverse transcription-PCR analysis of the expression of MMP-2, MMP-7, MMP-9, and MMP-13 mRNA. The leptin induced the transcriptional activation of MMP-13. **B**-**D.** PANC-1 or AsPC-1 cells were treated with leptin alone or in combination with the JAK2 inhibitor AG490 for 24 h. Untreated cells were used as controls. **B.** The cell lysates were subjected to a western blot analysis of the MMP-13 protein. The histogram shows the densitometric analysis of the bands; the data are expressed as the fold changes in the levels of MMP-13 with respect to β-actin. **C.** The MMP-13 enzyme activity in the culture supernatants was analyzed using collagen zymography. **D.** The MMP-13 mRNA level of the treated cells was also analyzed using quantitative reverse transcription-PCR. The leptin enhanced the MMP-13 expression and activity via the JAK2/STAT3 pathway. **E**, **F.** PANC-1 cells were infected with a recombinant lentivirus carrying the human MMP-13 gene (MMP-13-overexpressing cells, PANC-1-MMP13) or an empty virus (control cells, PANC-1-Lv). **E.** A western blot analysis of the expression of MMP-13 in the PANC-1-MMP13 and PANC-1-Lv cells. **F.** Both of the infected cells were subjected to an *in vitro* scratch assay (left panel) and a Matrigel-based invasion assay (right panel). The MMP-13 overexpression significantly enhanced the invasion, but not the migration, of the pancreatic cancer cells. **G**, **H.** PANC-1 or AsPC-1 cells were treated with leptin alone or in combination with the MMP-13 inhibitor CL82198. Untreated cells were used as controls. The treated cells were subjected to an *in vitro* scratch assay **G.** and a Matrigel-based invasion assay **H.**. The inhibition of MMP-13 attenuated the leptin-stimulated invasion of the pancreatic cancer cells. The data are presented as the mean ± SEM of 3-4 independent experiments. * *P* < 0.05, ** *P* < 0.01, compared with the control cells; NS *P* > 0.05, ^#^ *P* < 0.05, ^##^ *P* < 0.01, compared with the leptin-treated or MMP-13-overexpressing cells.

Leptin signaling has been reported to regulate the production of various MMPs, including MMP-2 [32, 33], MMP-7 [34], MMP-9 [33], and MMP-13 [35]. MMPs play an important role in tumor metastasis due to their ability to degrade the extracellular matrix [36]. To understand whether and which types of MMPs were involved in the pancreatic cancer cell invasion triggered by leptin, we analyzed the expression change of MMP-2, MMP-7, MMP-9 and MMP-13 before and after leptin treatment. As shown in Figure 3A, the treatment with leptin resulted in a significant increase in the MMP-13 mRNA expression in both the PANC-1 and AsPC-1 cells but did not change the mRNA levels of MMP-2, MMP-7 or MMP-9. At the protein level, there was also significantly increased expression of MMP-13 in response to the leptin treatment (Figure 3B). Furthermore, collagen zymography demonstrated a similar enhancement of the MMP-13 enzyme activity in the supernatants of the leptin-treated cells (Figure 3C). To test whether the activation of JAK2/STAT3 contributed to the upregulation of MMP-13, we applied the JAK2 inhibitor AG490 in the study. As shown in Figure 3B to 3D, blocking the JAK2/STAT3 pathway using AG490 significantly attenuated the leptin-induced enhancement of the MMP-13 expression and activity, suggesting that MMP13 is regulated by JAK2/STAT3 after leptin stimulation.

Although MMP-13 has been suggested to be linked to the metastatic potential of pancreatic cancer via immunohistochemistry analysis and cDNA array assay [37, 38], this has never been proven experimentally. To investigate whether MMP-13 can increase the mobility of pancreatic cancer cells *in vitro*, we overexpressed MMP-13 in PANC-1 cells (PANC-1-MMP13) using lentiviral vectors (Figure 3E). The overexpression of MMP-13 significantly promoted the invasion of the PANC-1 cells but did not affect their migration potential (Figure 3F). We further used the MMP-13 inhibitor CL82198 to confirm that leptin's action on the pancreatic cancer cells was mediated by MMP-13. As shown in Figure 3G and 3H, CL82198 significantly antagonized the increased invasion, but not the migration, induced by leptin in both the PANC-1 and AsPC-1 cells. Taken together, our data suggest that MMP-13 may serve as a downstream effector of the leptin -JAK2/STAT3 cascade responsible for pancreatic cancer cell invasion.

### Leptin promotes the growth and metastasis of pancreatic cancer *in vivo*

**Figure 4:**
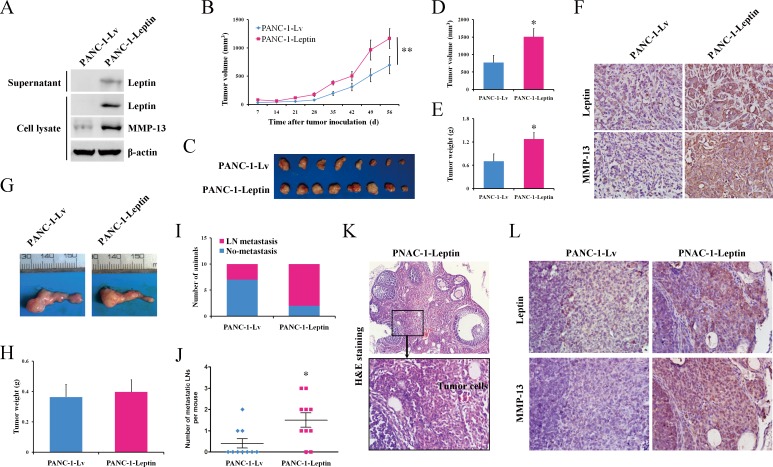
Leptin promotes tumor growth and metastasis to the lymph nodes in mouse models of human pancreatic cancer PANC-1 cells were infected with recombinant lentivirus carrying the human leptin gene (leptin-overexpressing cells, PANC-1-Leptin) or an empty virus (control cells, PANC-1-Lv). **A.** A western blot analysis of the expression of leptin and MMP-13 in the PANC-1-Leptin and PANC-1-Lv cells. Both of the infected cells were subcutaneously injected into nude mice (*n* = 8 per group). **B.** The tumor growth was monitored by measuring the tumor volume once a week following the implantation. **C.** The mice were euthanized when moribund (day 63), and the subcutaneous tumors were dissected and photographed. **D**, **E.** The comparison of the tumor volume **D.** and the tumor **E.** weight between the PANC-1-Lv and PANC-1-Leptin groups at the time of sacrifice. The leptin overexpression significantly promoted the growth of the subcutaneous pancreatic tumors. **F.** The representative immunohistochemical staining (original magnification, ×200) of leptin and MMP-13 in the subcutaneous tumors. The PANC-1-Lv and PANC-1-Leptin cells were also orthotopically injected into the pancreas of nude mice (*n* = 10 per group). **G.** The representative orthotopic pancreatic tumors were dissected at the time of euthanasia (day 53). **H.** The comparison of the orthotopic tumor weights between the PANC-1-Lv and PANC-1-Leptin groups. **I**, **J.** At the end of the experiment, the animals with pancreatic tumor metastasis to the celiac lymph node **I.** and the metastatic lymph node **J.** were counted. The leptin overexpression significantly promoted the lymph node metastasis of the pancreatic tumor. LN, lymph node. **K.** The representative H&E staining of the metastatic lymph node from the PANC-1-Leptin tumor-bearing mice. The boxed area in the upper panel (×100) is shown at a higher magnification in the lower panel (×400). **L.** The representative immunohistochemical staining (original magnification, ×400) of leptin and MMP-13 in the metastatic lymph nodes. The data are presented as the mean ± SEM. * *P* < 0.05, ** *P* < 0.01, compared with the control group.

To investigate the *in vivo* impact of leptin on pancreatic cancer, we established a PANC-1 pancreatic cancer cellline stably overexpressing leptin (PANC-1-Leptin). Western blot analyses demonstrated the increased concentration of leptin in both the cell lysates and supernatants of the cultured PANC-1-Leptin cells (Figure 4A), confirming the exogenous expression and secretion of leptin in the PANC-1-Leptin cells. Meanwhile, the overexpression of leptin was coupled with the upregulation of MMP-13 (Figure 4A), and it significantly enhanced the cell migration and invasion *in vitro* (Supplementary Figure S1). The PANC-1-Leptin and the control PANC-1-Lv cells were then inoculated subcutaneously into nude mice, and the tumor growth was monitored once per week. The leptin-overexpressing xenografts had a significantly faster growth rate than the controls (Figure 4B). At the time of euthanasia, the mean volume of the leptin-overexpressing tumors was significantly larger than that of the tumors in the control group (Figure 4C and 4D). Similar to the tumor volume, the tumor weight was increased by 81.6% in the leptin-overexpressing tumors (Figure 4E). In both the leptin-overexpressing cells (Figure 4A) and tumors (Figure 4F), MMP-13 was consistently upregulated.

To further confirm the above observation, the PANC-1-Leptin and PANC-1-Lv cells were orthotopically implanted in the pancreas of nude mice. The mice were euthanized at 53 days post-inoculation, and we did not find any significant differences in the orthotopic tumor weights between the groups (Figure 4G and 4H). However, interestingly, more mice bearing leptin-overexpressing tumors (8 out of 10) developed celiac lymph node metastases compared to the control mice (3 out of 10; Figure 4I). And the average number of metastatic lymph nodes in the leptin-overexpressing mice was also significantly greater than in the control mice (Figure 4J and 4K). However, we did not observe metastasis to the liver, lung, or spleen in either group. In addition, we detected increased expression of MMP-13 in the metastatic tumors overexpressing leptin (Figure 4L), suggesting an important role of MMP-13 in leptin-induced cancer progression.

### The tumoral expression of Ob-Rb and MMP-13 is associated with lymph node metastasis in pancreatic cancer patients

**Figure 5:**
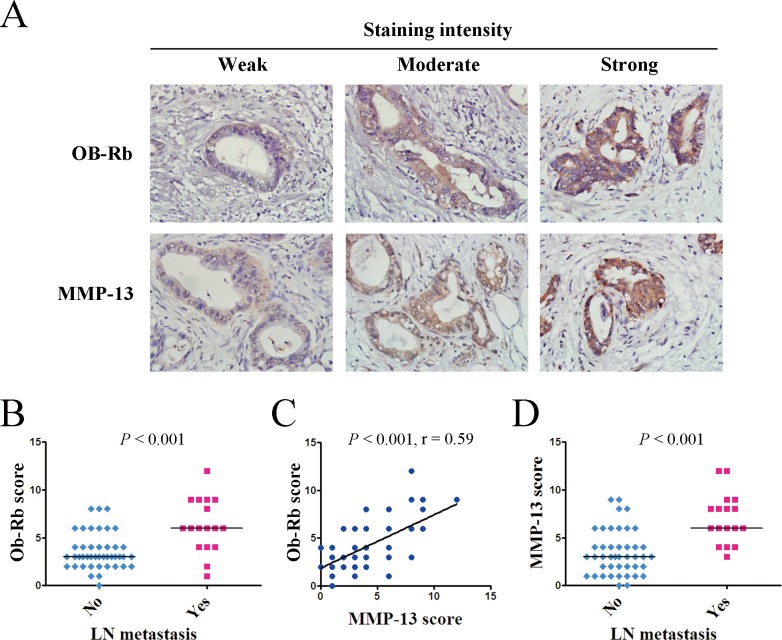
The intratumoral expression of the functional leptin receptor Ob-Rb correlates with the MMP-13 expression and lymph node metastasis in pancreatic cancer patients **A.** The representative immunohistochemical staining (original magnification, ×400) of the long form of the leptin receptor Ob-Rb and MMP-13 in human pancreatic cancer tissues. **B.** The Ob-Rb expression scores for the pancreatic cancer patients with or without lymph node metastasis. **C.** The analysis of the correlation between the Ob-Rb and MMP-13 expression in 60 pancreatic cancer tissues. The analysis was performed using the Spearman rank-correlation test. The Ob-Rb expression showed a positive association with the MMP-13 expression in the human pancreatic cancer tissues. **D.** The MMP-13 expression score for the pancreatic cancer patients with or without lymph node metastasis. **B**, **D.** The statistical analyses were performed using the Wilcoxon rank sum test. The patients with lymph node metastasis had significantly higher expression scores for both OB-Rb and MMP-13 than those without lymph node metastasis. The bar represents the median expression score.

To further understand the relationship between the leptin/MMP-13 axis and human pancreatic cancer development, we performed immunohistochemical staining of Ob-Rb and MMP-13 in 60 pancreatic cancer tissue samples (Figure 5A). Our analysis showed that the expression of Ob-Rb (represented as an expression score) in the pancreatic cancer samples was significantly stronger in the patients with lymph node metastasis than in those without lymph node metastasis (*P* < 0.001, Figure 5B & Table 1), and it tended to be stronger in the patients with advanced-stage pancreatic cancer than in those with the early stages of the disease (*P* = 0.09, Table 1). Moreover, as shown in Figure 5C, the Ob-Rb levels were significantly correlated with the MMP-13 levels in the pancreatic cancer tissues (*r* = 0.59, *P* < 0.001). Consistently, we also found the association of MMP-13 expression with lymph node metastasis and the pathological stage (Figure 5D & Table 1). These data support the role of the leptin/MMP-13 axis in pancreatic cancer metastasis.

**Table 1:** Ob-Rb or MMP-13 expression and clinicopathological features of pancreatic cancer patients

	Ob-Rb		MMP-13	
Parameters	Expression score (median, range)	*P* value	Expression score (median, range)	*P* value
Gender		0.263		0.509
Male (n = 27)	4, 0-12		3, 1-12	
Female (n = 33)	3, 1-9		4, 0-12	
Age		0.772		0.934
≤ 60 (n = 25)	4, 1-8		4, 0-9	
> 60 (n = 35)	3, 0-12		4, 0-12	
Location		0.922		0.615
Head (n = 33)	4, 0-9		4, 0-12	
Body/tail (n = 27)	4, 1-12		4, 1-12	
Size (cm)	0.652		0.374	
≤ 3 (n = 31)	4, 0-9		4, 0-12	
> 3 (n = 29)	3, 1-12		4, 0-8	
Histological grade		0.527^#^		0.610^#^
Grade 1 (n = 8)	6, 2-8		5, 1-9	
Grade 2 (n = 29)	4, 0-9		4, 0-12	
Grade 3 (n = 23)	3, 1-12		4, 1-8	
TNM stage	0.090			0.073
I+II (n = 52)	3, 0-9		3.5, 0-12	
III+IV (n = 8)	6, 2-12		8, 0-12	
LN metastasis		<0.001^*^		<0.001^*^
No (n = 43)	3, 0-8		3, 0-9	
Yes (n = 17)	6, 1-12		6, 3-12	

# *P* was calculated by Kruskal-Wallis test

* Statistically significant (*P* < 0.05).

## DISCUSSION

Substantial evidence indicates that obesity and diabetes mellitus are two risk factors for pancreatic cancer [4-6]. However, the molecular connection between metabolic diseases and pancreatic cancer is obscure. Leptin, a 16-kDa protein encoded by the *ob* gene, is predominantly produced by adipocytes [13]. It was originally described as a circulating hormone involved in feeding behavior and energy homeostasis through its actions on the hypothalamus, where Ob-Rs are highly expressed [39, 40]. Later, Ob-Rs were found to have a wide distribution in different tissues [16], suggesting that leptin may have numerous peripheral effects. Indeed, leptin has since been found to be able to regulate numerous biological processes, including inflammation, angiogenesis, and proliferation [15-17]. In this study, we focused our interests on leptin's effect on pancreatic cancer. We demonstrated that the leptin receptor Ob-Rb is present in pancreatic cancer cells, and the activation of Ob-Rb can enhance the invasion of pancreatic cancer via upregulating MMP-13 production. These findings highlight the potential of leptin/Ob-Rb as a novel therapeutic target for pancreatic cancer.

The deregulation of leptin and Ob-Rs has been shown in various cancers [17]. As for pancreatic cancer, there is a lack of convincing clinical investigations on the relationship between circulating leptin levels and cancer, until now. From the existing results, it appears that patients with pancreatic cancer had a lower concentration of circulating leptin compared to the controls [41-43]. However, it should be noted that all of the available studies are cross-sectional studies, where the circulating leptin levels were evaluated after the diagnosis of pancreatic cancer. Because pancreatic cancer patients usually have pathological weight loss or cachexia, the decreased leptin concentrations may be secondary to the loss of body fat mass [42]. The real correlation between the circulating leptin and pancreatic cancer still requires data from prospective cohort studies. On the converse, it is also possible that the carcinogenic effect of leptin on the pancreas is induced by an overabundance of local rather than systemic leptin [44]. For these reasons, we focused our study on the leptin receptor instead of leptin itself.

The leptin receptor has been reported to be necessary for maintaining the cancer cell's stem cell-like properties in triple-negative breast cancer cells [45]. The aberrant expression of Ob-Rb in cancer cells has been suggested to be a useful prognostic marker in ovarian, breast, and endometrial cancer [46-48]. However, limited data are available on the state of Ob-Rb and its clinical relevance in pancreatic cancer. Therefore, our findings presented in this study are novel and interesting. Given that leptin receptor antagonists effectively inhibit breast cancer in mouse models [49], it is promising that utilizing a synthetic analog or monoclonal antibody as a leptin receptor antagonist may become a new strategy for the treatment for pancreatic cancer.

Leptin has been reported to have different effects on cancer cell proliferation. In most cancers, such as breast, prostate, lung and colorectal cancer, leptin acts as a growth factor that has the capability to promote cancer cell proliferation [17, 18]. However, in one *in vitro* study, leptin was shown to inhibit the growth of pancreatic cancer cells [50]. Such an inhibitory effect of leptin on pancreatic cancer was not observed in our study. In our *in vitro* experiment, the growth curves were almost identical between the leptin-treated or non-treated groups in both the PANC-1 and AsPC-1 cells. The *in vivo* experiments confirmed that leptin has no inhibitory effect on pancreatic cancer. In a subcutaneous transplanted tumor model, leptin even promoted the growth of cancer xenografts. The factors contributing to this discrepancy are not clear from the current data. A possible explanation is that the leptin concentration we and many others [22-24, 26] have used to treat the cells was 50-100 ng/ml, while in the study showing that leptin inhibited pancreatic cancer cell growth [50], the authors used 0.4-4 ng/ml of leptin for stimulation. Because the physiological levels of circulating leptin in humans is approximately 4 ng/ml, whether leptin has a divergent function on cell growth at elevated or reduced pathological concentrations is an interesting issue and warrants further investigation. It will aid in the understanding of the differential oncogenic impacts of hyperleptinemia during the pre-cancer stage and hypoleptinemia during the advanced stages of pancreatic cancer.

The impact of leptin on cancer metastasis has never been investigated in pancreatic cancer. Although the leptin-stimulated enhancement of cell migration and invasion has been reported before in various cancers, the evidence was obtained from the *in vitro* experiments such as wound healing or Transwell assay [22, 26, 32, 34, 35]. Thus, our *in vivo* results were novel not only for pancreatic cancer, but also for other types of cancers. Another novel finding of this study is that MMP-13 may play an important role in leptin-induced pancreatic cancer metastasis. Human MMP-13, also known as collagenase-3, is a matrix metalloproteinase originally identified in breast carcinomas [51]. Recent studies have revealed that this enzyme is also produced by a variety of malignant tumors, including head and neck, breast and colorectal cancer [52-54]. In all of the cases, the expression of MMP-13 is associated with aggressive tumors. Pancreatic cancer is known to be an extremely lethal neoplasm that has a high potential for invasion and metastasis. In the present study, we compared the expression levels of MMP-2, MMP-7, MMP-9 and MMP-13 in pancreatic cancer cells with and without leptin treatment. We found that only MMP-13, but not MMP-2, MMP-7 or MMP-9, was altered following the leptin stimulation. The leptin-induced MMP-13 expression was blocked when a JAK2/STAT3 inhibitor was introduced into the experiments, confirming that MMP-13 is a responsive downstream molecule of leptin. In addition, in both mouse transplanted pancreatic cancer and human clinical pancreatic cancer tissues, we observed a correlation between Ob-Rb and MMP-13. Therefore, the conclusion that leptin regulates MMP-13 in pancreatic cancer is solid. Previously, leptin was reported to be able to increase the expression of MMP-2, MMP-7 or MMP-9 in different cancer cells [32-34], as well as the expression of MMP-13 in glioma cells [35]. This suggested that leptin's regulation of MMPs is in a tissue-specific manner. In glioma cells, the up-regulation of MMP-13 by leptin was mediated through p38 MAP kinase and NF-κB pathway [35], while in pancreatic cancer cells, we found that the expression of MMP-13 was regulated through JAK2/STAT3 signaling pathway in response to leptin stimulation. Recently, Tan et al compared the global gene expression of two pancreatic cancer cell lines with different potentials for invasion-metastasis using cDNA microarray technique [38]. They found that the expression of MMP-13 was markedly increased (30-fold) in the highly invasive/metastatic cells compared with the weakly invasive/metastatic cells [38]. The translational value of MMP-13 in the diagnosis, prognosis and treatment of pancreatic cancer is definitely worth further exploration.

The limitation of this study is that we did not simultaneously test the leptin expression using immunohistochemistry in the pancreatic cancer tissues due to the secretory nature of leptin, which makes quantitative analysis a challenge. Therefore, it remains to be determined whether the action of leptin in pancreatic cancer is exerted via an autocrine, paracrine or endocrine mode.

Taken together, this study provides the *in vitro* and *in vivo* evidence, for the first time, that leptin signaling contributes to the invasion and metastasis of pancreatic cancer. It also suggests a novel JAK2/STAT3/MMP-13 axis by which leptin exerts its action on pancreatic cancer metastasis (schematic diagram in Supplementary Figure S2). This study suggests leptin signaling as an attractive target for the treatment of pancreatic cancer, especially for patients with metabolic disorders. Recently, studies from Blagosklonny's group have found that intermittent administration of rapamycin could prevent weight gain and reduce peripheral leptin level in mice on high-fat diet without changing metabolic parameters such as insulin, glucose, triglycerides and insulin-like growth factor 1 [55, 56]. Another study has shown that in a rat model of obesity, rapamycin was able to normalize elevated serum leptin by alleviating obesity and decreasing leptin synthesis in white adipose tissues [57]. Therefore, rapamycin may be used as an anti-leptin agent for the prevention and intervention against obesity-related pancreatic cancer. Interestingly, a pilot study has already shown that rapamycin could effectively inhibit pancreatic cancer growth in diet-induced obese mice [58]. Although the tumor suppressive effect of rapamycin was thought to be mediated through PI3K/mTOR signaling pathway, our current findings suggest that downregulation of leptin levels may also contribute to the rapamycin-induced pancreatic cancer suppression.

## MATERIALS AND METHODS

### Reagents and antibodies

The recombinant human leptin was purchased from R&D Systems (Minneapolis, MN). The JAK2 inhibitor AG490 was obtained from Cell Signaling Technology (Beverly, MA), and the MMP-13 inhibitor CL82198 was obtained from Sigma (St. Louis, MO). The following antibodies were used in the study: the antibody for the long form of the leptin receptor (Ob-Rb) was purchased from Proteintech Group (Chicago, IL); the STAT3 and phosphorylated STAT3 (pSTAT3^Try705^) antibodies were purchased from Cell Signaling Technology; the MMP-13 antibody was purchased from Abcam (Cambridge, MA); the leptin antibody was purchased from BioVendor (Brno, Czech Republic); and the β-actin antibody was purchased from Sigma.

### Cell culture and treatment

The human pancreatic cancer cell lines PANC-1 and AsPC-1 were acquired from the American Type Culture Collection (Manassas, VA) and maintained in DMEM and RMPI-1640 medium (Hyclone, Logan, UT) supplemented with 10% fetal bovine serum (Invitrogen, Carlsbad, CA), respectively. For the treatment, the human pancreatic cancer cells were serum starved for 16 h and were then treated with recombinant human leptin at 100 ng/ml in the absence or presence of AG490 (100 μmol/l) or CL82198 (10 μg/ml) for the indicated durations.

### RNA isolation and RT-PCR

The total cellular RNA was extracted using TRIzol reagent (Invitrogen) and was subjected to reverse transcription using the FastQuant RT kit with gDNase (Tiangen Biotech, Beijing, China). The synthesized cDNA was used as a template for the semiquantitative PCR detection of the mRNA expression of Ob-Rt and Ob-Rb as previously described [22]. A quantitative real-time PCR analysis of the mRNA expression of the MMPs was performed using the FastStart Universal SYBR Green Master kit (Roche Diagnostics, Mannheim, Germany) using the StepOne Plus Real-time PCR system (Applied Biosystems, Foster City, CA). The relative expression levels of the target genes were calculated following normalization against a reference gene (*Gapdh*). The primers for the semiquantitative or quantitative PCR are provided in Supplementary Table S1.

### Western blot

Whole-cell lysates were prepared using RIPA buffer (Cell Signaling Technology), and the proteins were quantified using the Pierce BCA Protein Assay kit (Thermo Scientific, Rockford, IL) according to the manufacturer's instructions. Equal amounts of proteins were separated using SDS-PAGE electrophoresis and were blotted onto PVDF membranes. After blocking with SuperBlock blocking buffer (Thermo Scientific), the blots were probed with the antibodies described above. The immunodetection was performed using Pierce SuperSignal West Pico chemiluminescent substrate (Thermo Scientific).

### Cell proliferation assay

Approximately 5 × 10^3^ serum-starved cells were cultured in the presence of leptin for 24, 48, or 72 h in 96-well tissue culture-plates. The cell proliferation was assessed using the CellTiter 96 MTS Assay (Promega, Madison, WI) according to the vender's instructions. The 490-nm absorbance was measured after a 2-hour incubation with the MTS reagent.

### Cell migration assay (scratch assay)

The cell migration was measured using a scratch wound-healing assay. After 16 h of serum starvation, the confluent cell monolayers were wounded by scratching with a sterile pipette tip. Then, the cells were washed with serum-free medium to remove any debris and were subsequently treated with leptin in the absence or presence of AG490 or CL82198. At time 0 and after 12 or 24 h, the plates were examined and photographed using a phase-contrast microscope. The cell migration was quantitated by counting the number of cells that had migrated into the wound. The data were expressed as the relative changes in the cell migration.

### Cell invasion assay

For the *in vitro* tumor cell invasion studies, a Matrigel-based invasion assay was performed using 8-μm pore-size culture inserts (BD Biosciences, Bedford, MA) coated with Matrigel (BD Biosciences). The control cells or the leptin-treated cells were suspended in serum-free medium and seeded into upper inserts at 1 × 10^5^ cells per insert, and a 10% FBS medium was used as a chemoattractant in the lower chamber. In some of the experiments, the cells were treated with AG490 or Cl82198 along with the leptin. After 24 h of incubation, the cells remaining on the upper surface of the insert membrane were gently removed using a sterile cotton swab. The cells that had invaded through the Matrigel and adhered to the bottom of the insert membrane were fixed and stained with 0.1% crystal violet in 20% methanol. The cell invasion was quantitated by counting the number of invaded cells. The data were expressed as the relative changes in the cell invasion.

### Collagen zymography

Samples of the supernatant medium that were conditioned using different cell cultures were centrifuged to remove any cell debris and were then concentrated using Amicon Ultra 10K Centrifugal Filters (EMD Millipore, Billerica, MA). Equal volumes of each sample were subjected to collagen zymography using an MMP Zymography (MMP-1/MMP-13) Assay Kit (GENMED, Shanghai, China) according the manufacturer's protocol. The location of the collagenolytic activity was visualized as clear bands against a blue background.

### Lentivirus vector construction and transduction

The full-length human leptin or MMP-13 genes (Genechem, Shanghai, China) were subcloned into the expression lentivector pCDH-CMV-MCS (System Biosciences, Mountain View, CA) between the *Nhe*I and *Sal*I sites. Packaging of the lentivirus was performed using the pPACKH1 Lentivector Packaging kit (System Biosciences) according to the manufacturer's instructions. The infectious lentiviral particles were concentrated via centrifugation with an Amicon Ultra 100K Centrifugal Filter (EMD Millipore). The PANC-1 cells were then transduced with the recombinant lentivirus carrying the human leptin or MMP-13 gene to generate stable transduced PANC-1-Leptin or PANC-1-MMP13 cells. The PANC-1 cells were also infected with an empty virus and used as control cells (PANC-1-Lv). The primers for cloning are listed in Supplementary Table S1.

### Animal experiment

The male athymic BALB/c nude mice (5-6 weeks old) used in this study were maintained under specific pathogen-free conditions, and were manipulated in accordance with the ethical guidelines provided under the protocols approved by the Medical Experimental Animal Care Commission at Shanghai Jiaotong University.

For the subcutaneous model of pancreatic cancer, the mice were randomly divided into groups and were subcutaneously injected in their right flanks with 3 × 10^6^ PANC-1-Lv or PANC-1-Leptin cells. The tumor growth was monitored once a week and the tumor volume was calculated according to the following formula: volume = π/6 × width^2^ × length. The mice were sacrificed when moribund (day 63), and the tumors were excised and weighed.

For the orthotopic models of pancreatic cancer, the tumor cells were injected into the mouse pancreases according to a previously described method [59]. Briefly, the animals were anesthetized with an intraperitoneal injection of pelltrobarbitalum natricum. A small incision was made and 1 × 10^6^ PANC-1-Lv or PANC-1-Leptin cells suspended in 50 μl of a Matrigel mixture (1:1 volume) were injected into the head/neck region of the pancreas. At the end of the experiment (day 53), the mice were euthanized. The tumor-bearing pancreases were excised from each mouse, and the primary tumors were then identified and weighed. The other organs, including the liver, spleen, lung, and celiac LNs were checked for metastasis. The metastatic LNs were counted and confirmed histologically.

### Patients and pancreatic cancer tissues

A total of 60 pancreatic cancer tissue specimens were collected from patients who underwent surgical treatment at the East Surgery Department of Renji Hospital (Shanghai, China) between 2012 and 2013. The 60 pancreatic cancer patients, none of whom had received chemotherapy prior to the surgery, included 27 males and 33 females (median age 63 years, ranging from 37 to 91 years). All of the tissue specimens were routinely fixed in 4% phosphate-buffered neutral formalin and were embedded in paraffin. At least one pathologist examined the H&E-stained sections to ensure the presence of >85% tumor cells. The use of the tissue specimens in this study was approved by the Ethics Committee of Renji Hospital, and informed consent was obtained from each patient.

### Histological analyses

The tissue samples were fixed in 4% phosphate-buffered neutral formalin (for at least 72 h), embedded in paraffin, and sectioned at 5 μm. The sections were stained with H&E as per standard procedures. For the immunohistochemical staining, the sections were incubated with an anti-leptin antibody (1:400) or an anti-MMP-13 antibody (1:200) at 4°C overnight. The primary antibodies were detected using the Impress Universal kit (Vector Laboratories, Burlingame, CA) with NovaRed (Vector Laboratories) as a substrate. The human pancreatic cancer tissue sections were blindly examined and scored concurrently by two observers. The intensity of the immunostaining was scored as 0 (negative), 1 (weak), 2 (moderate), or 3 (strong). Additionally, the percentage of positive tumor cells was scored as 0 (no stained cells), 1 (< 25%), 2 (26-50%), 3 (51-75%), or 4 (76-100%). The final expression scores (ranging from 0 to 12) for each sample were determined as the product of the intensity score and the percentage score.

### Statistical analysis

The data were presented as the mean ± SEM unless indicated otherwise. The statistical analyses were performed using SPSS software version 19.0 (SPSS Inc., Chicago, IL). The significance of the differences between groups was assessed using Student's *t* test (for the normally distributed variables) or the Wilcoxon rank sum test (for the non-normally distributed variables) unless otherwise noted, and the level of significance was set at 0.05 for all of the analyses.

## SUPPLEMENTARY MATERIALS FIGURES AND TABLE


