# Supplementary material for: Leptin signaling enhances cell invasion and promotes the metastasis of human pancreatic cancer via increasing MMP-13 production
Source: Oncotarget. 2015 Apr 19;6(18):16120–34. doi: 10.18632/oncotarget.3878 (PMC4599260; doi:10.18632/oncotarget.3878)
Supplement: Supplementary file 1 [file oncotarget-06-16120-s001.pdf]

Leptin signaling enhances cell invasion and promotes the metastasis of human pancreatic cancer via increasing MMP-13 production

Supplementary Material

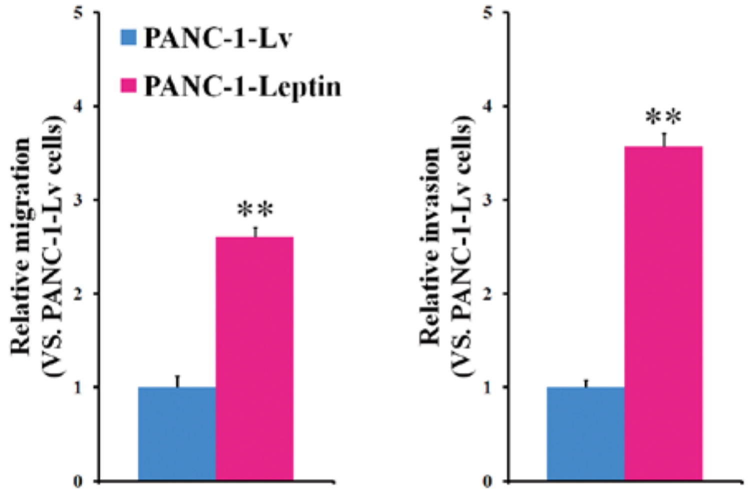

Supplementary Figure S1: Leptin overexpression promotes the migration and invasion of PANC-1 cells. PANC-1 cells were infected with the recombinant lentivirus carrying human leptin gene (leptin-overexpressing cells, PANC-1-Leptin) or empty virus (control cells, PANC-1-Lv). The infected cells were subjected to an *in vitro* scratch assay (left panel) and Matrigel-based invasion assay (right panel). Leptin overexpression significantly enhanced both the migration and invasion of the PANC-1 cells. Data are presented as mean ± SEM of 3 independent experiments. ** $P < 0.01$, compared with control cells.

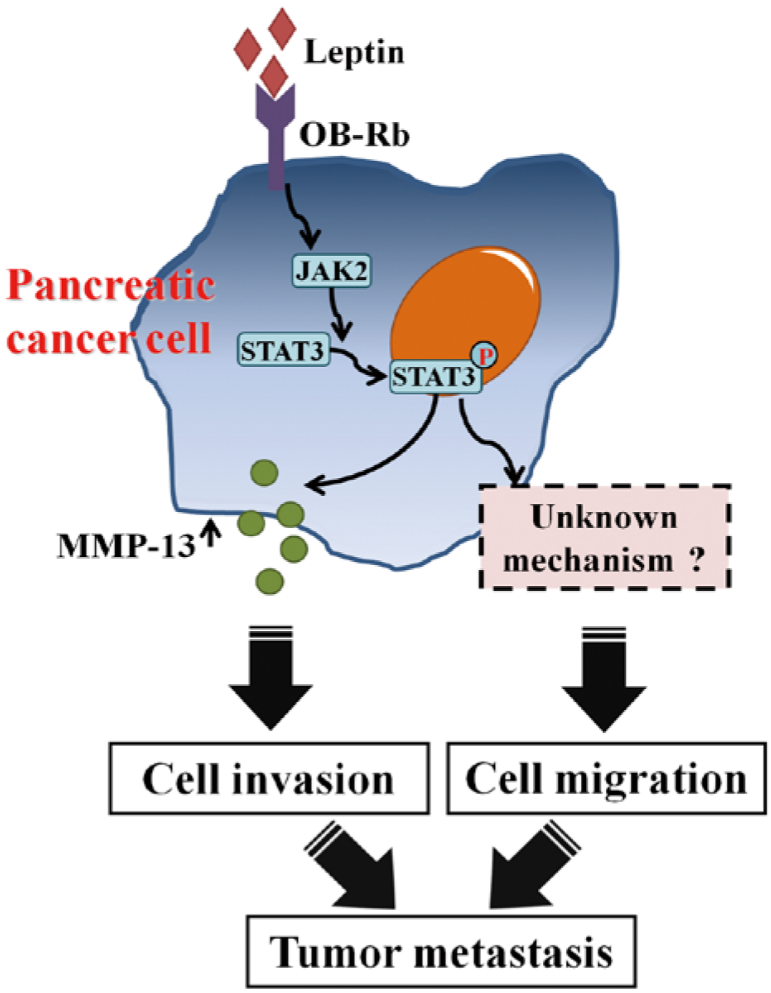

Supplementary Figure S2: Proposed mechanisms of leptin-induced enhancement of pancreatic cancer metastasis. The binding of leptin to its receptor Ob-Rb on pancreatic cancer cells activates JAK2/STAT3 signaling, which enhances cell invasion potential via increasing the expression and activity of matric metalloproteinase MMP-13. The leptin-induced activation of JAK2/STAT3 pathway also enhances the migration potential of pancreatic cancer cells through unknown mechanisms. As a consequence, leptin promotes the metastasis of pancreatic cancer.

Supplementary Table S1: Primers used in this study

Name	Primer sequence
For semiquantitative PCR	
Ob-Rb forward	5’-TCACCCAGTGATTACAAGCT
Ob-Rb reverse	5’-CTGGAGAACTCTGATGTCCG
Ob-Rt forward	5’-CATTTTATCCCCATTGAGAAGTA
Ob-Rt reverse	5’-CTGAAAATTAAGTCCTTGTGCCCAG
18S RNA forward	5’-GAGGGAGCCTGAGAAACGG
18S RNA reverse	5’-GTCGGGAGTGGGTAATTTGC
For quantitative PCR	
MMP-2 forward	5’-TCTTCCCTCGCAAGCCCAAGT
MMP-2 reverse	5’-GAAGGCACGAGCAAAGGCATCA
MMP-7 forward	5’-TGCGACTCACCGTGCTGTGT;
MMP-7 reverse	5’-GTCCTGAGCCTGTTCCCACTGT
MMP-9 forward	5’-ACCCTTGTGCTCTTCCCTGGAG;
MMP-9 reverse	5’-TCTCTGCCACCCGAGTGTAACC
MMP-13 forward	5’-GTCCTGGCTGCCTTCCTCTTCT;
MMP-13 reverse	5’-CGCTCTGCAAACTGGAGGTCTT
GAPDH forward	5’-ATCCTGGGCTACACTGAGCACC
GAPDH reverse	5’-AAGTGGTCGTTGAGGGCAATGC
For cloning	
Leptin-fl forward	5’-GATGCTAGCGCCACCATGCATCCAGGGGTCCTG;
Leptin-fl reverse	5’-CATGTCGACTTAACACCACAAAATGGA
MMP-13-fl forward	5’-GATGCTAGCGCCACCATGCATTGGGGAACCCTGTGCG
MMP-13-fl rewerse	5’-CATGTCGACTCAGCACCCAGGGCTGAGGT
